# Rose Bengal diacetate-mediated antimicrobial photodynamic inactivation: potentiation by potassium iodide and acceleration of wound healing in MRSA-infected diabetic mice

**DOI:** 10.1186/s12866-024-03401-6

**Published:** 2024-07-05

**Authors:** Danfeng Wei, Michael R Hamblin, Hao Wang, Reza Fekrazad, Chengshi Wang, Xiang Wen

**Affiliations:** 1 Department of Dermatology, West China Hospital, Sichuan University, No.37 Guo Xue Alley, Chengdu, 610041 China; 2 Laboratory of Dermatology, Clinical Institute of Inflammation and Immunology, Frontiers Science Center for Disease-related Molecular Network West China Hospital, Sichuan University, Chengdu, 610041 China; 3 Laser Research Centre, Faculty of Health Science, University of Johannesburg, Doornfontein, 2028 South Africa; 4 Radiation Sciences Research Center, Laser Research Center in Medical Sciences, AJA University of Medical Sciences, Tehran, Iran; 5 International Network for Photo Medicine and Photo Dynamic Therapy (INPMPDT), Universal Scientific Education and Research Network (USERN), Tehran, Iran; 6 Department of Endocrinology and Metabolism, Center for Diabetes and Metabolism Research, West China Hospital, Sichuan University, Chengdu, 610041 China

**Keywords:** Antimicrobial photodynamic inactivation, Methicillin-resistant *staphylococcus aureus*, Rose bengal diacetate, Potassium iodide potentiation, Diabetic mouse infected wound

## Abstract

Previous studies have shown that antimicrobial photodynamic inactivation (aPDI) can be strongly potentiated by the addition of the non-toxic inorganic salt, potassium iodide (KI). This approach was shown to apply to many different photosensitizers, including the xanthene dye Rose Bengal (RB) excited by green light (540 nm). Rose Bengal diacetate (RBDA) is a lipophilic RB derivative that is easily taken up by cells and hydrolyzed to produce an active photosensitizer. Because KI is not taken up by microbial cells, it was of interest to see if aPDI mediated by RBDA could also be potentiated by KI. The addition of 100 mM KI strongly potentiated the killing of Gram-positive methicillin-resistant *Staphylocccus aureus*, Gram-negative *Eschericia coli*, and fungal yeast *Candida albicans* when treated with RBDA (up to 15 µM) for 2 hours followed by green light (540 nm, 10 J/cm^2^). Both RBDA aPDI regimens (400 µM RBDA with or without 400 mM KI followed by 20 J/cm^2^ green light) accelerated the healing of MRSA-infected excisional wounds in diabetic mice, without damaging the host tissue.

**Supplementary Information:**

The online version contains supplementary material available at 10.1186/s12866-024-03401-6.

## Introduction

Treatment of wounds in patients with systemic diseases, such as diabetes mellitus, is a significant clinical problem. Infections and delayed wound healing are regularly seen. Antibiotics have been widely used in wound infections, but the rise of antibiotic resistance is now a serious threat to public health. *Staphylococcus aureus* is a leading cause of skin and soft tissue infections (SSTI), while life-threatening conditions such as bacteremia and sepsis can also occur [1]. Among the antibiotic-resistant “superbugs”, methicillin-resistant *S. aureus* (MRSA) was described as the world’s most dangerous pathogen by the World Health Organization (WHO) [2]. Infection by MRSA is one of the main reasons for chronic non-healing wounds in diabetic patients [3], who sometimes require amputations with long hospitalization stays and can suffer high mortality from sepsis [4]. So, it is urgent to develop alternative antimicrobial strategies [5, 6]. Photodynamic therapy (PDT) refers to a photochemical reaction between non-toxic photosensitizers (PS) and light in the presence of oxygen, which was originally developed as a cancer treatment [7]. Antimicrobial photodynamic inactivation (aPDI) is the microbiological application of PDT, which may be an effective alternative to conventional anitibiotics in localized infections, since preliminary studies have shown that aPDI can improve infected wound healing both in mice and humans [8, 9].

Rose Bengal (RB) is a xanthene dye, among the most well-characterized PS, and is highly effective in light-mediated killing of Gram-positive bacteria, while it is largely inactive in killing Gram-negative bacteria [10]. Gram-positive bacterial cell walls are highly permeable, while Gram-negative bacteria are mostly impermeable to PS with anionic or neutral charges [11]. The intracellular accumulation of PS depends on their structure, physicochemical properties, charge and solubility [12]. Rose Bengal diacetate (RBDA) also known as Rose Bengal acetate (RBAc) is a lipophilic derivative of RB with two acetate groups [13]. The acetate groups allow the photosensitizer to be easily internalized by bacterial (as well as host) cells. When inside the cell, the acetate ester groups are recognized by a variety of esterase enzymes and hydrolyzed, thus liberating the free RB to act as an active PS. This is important since the intracellular localization of RB is crucial for bacterial killing [14]. RBDA-PDT has been shown to exert a more effective phototoxic effect against tumor cells compared to free RB [13], but reports of the use of RBDA-PDT for bacterial killing have so far been limited [14].

We previously found that the non-toxic inorganic salt potassium iodide (KI) showed a remarkable potentiating effect on RB-mediated aPDI using 540-nm green light against Gram-positive, Gram-negative bacteria, and fungal yeast in vitro, and also in vivo using a mouse model of wound infection [10]. The present study aimed to explore whether the aPDI effects of RBDA would also be potentiated by the addition of KI, and could this treatment promote healing of infected wounds in diabetic mice.

## Materials and methods

### Chemicals and reagents

Rose Bengal diacetate (RBDA, Santa Cruz Biotechnology, Dallas, TX, USA), KI (Zhongtian Fine Chemical, Qingdao, China), Agar (Sangon Biotech, Shanghai, China), Bacterial reactive oxygen species detection kit (Bestbio Biotechnology, Shanghai, China). Single Oxygen Sensor Green, Live/DeadTM BacLightTM Bacterial Viability Kit, Anti-IL-6 antibody, Anti-IL-1 beta antibody, Anti-Ki67 antibody (Thermo Fisher Scientific, Waltham, MA, USA), Bacteria DNA Kit (Tiangen Technology, Beijing, China). PAGE Gel Rapid preparation Kit (YaMei, Shanghai, China) and Coomassie Brilliant Blue (Beyotime Biotechnology, Shanghai, China).

### Cells and culture conditions

The following microbial strains were used: *MRSA Staphylococcus aureus* USA300 (ATCC BAA-1717), *Escherichia coli* (ATCC 25,922) and *Candida albicans* SC5314 (ATCC MYA-2876) were all purchased from American Type Culture Collection, VA, U.S.A and stored in the Department of Dermatology, West China Hospital, Sichuan University.

The bacteria were cultured in brain-heart infusion (BHI) broth at 37 °C, 220 rpm. *C. albicans* was cultured in yeast extract peptone dextrose medium (YPD) at 30 °C, 200 rpm. The ultraviolet absorption of the bacterial suspension at 600 nm was measured to obtain a suspension with a bacterial count of 6 × 10^7^ CFU/mL. A solid medium was prepared by addition of 1% agar powder to liquid BHI, and cultured in a dark incubator at 37 °C.

### Light source

A green light source consisted of a white lamp with a band-pass filter probe (wavelength, 540 ± 15 nm, Changchun Ocean Electro-optics, Changchun, China). The diameter of the light spot was 4 cm, and it could deliver 10 J/cm^2^ of energy by illuminating 4 wells of a 96-well plate for 8 min. Light power was measured with a power meter.

### Phototoxicity assay in vitro

RBDA was dissolved in methanol to form a 20 mM solution. For aPDI experiments, suspensions of bacteria (6 × 10^8^ CFU/mL) and *C. albicans* (6 × 10^7^ CFU/mL) in PBS were incubated with different concentrations of RBDA (0.1, 0.2, 0.5 and 1µM with MRSA; 8, 10, 12 and 14 µM with *E. coli*; 10 and 15 µM with *C. albicans*) with or without 100 mM KI in the dark for 1, 2 and 4 h at 37 °C. Then the bacterial suspension was placed in a 96 -well plate and exposed to 10 J/cm^2^ of green light. The experiment was divided into blank control group (no light, no RBDA), drug control group 1 (RBDA, no light), drug control group 2 (KI, no RBDA, no light), drug control group 3 (RBDA plus KI, no light), experimental group 1 (RBDA plus light), experimental group 2 (RBDA plus 100 mM KI plus light). After 8 min of light irradiation, the bacterial suspension was serially diluted to 10^− 5^ times the original density, and then the CFUs were calculated by means of drop plate culture counting, and each experiment was repeated three times. The number of logs (base 10) killed (KL) was used to measure the killing rate, N_0_ was the number of bacteria before aPDI, and Nt was the number of bacteria after aPDI.

The LIVE/DEAD BacLight Bacterial Viability Kit was also used to visualize the bacterial survival after RBDA-aPDI. Propidium-Iodide (PI) can stain the DNA of dead cells while SYTO9 is able to stain the DNA both in living and dead cells. The cells were treated as described above and co-stained with PI and SYTO9 in equal proportions in the dark for 15 min. Bacterial fluorescence was observed under a fluorescence inverted microscope with excitation wavelength of 488 nm and emission of 535 nm.

### Scanning electron microscopy

The MRSA cells after aPDI were washed with PBS and resuspended with electron microscopy fixative. Then the cells were fixed at room temperature for 2 h, and transferred to 4 °C for storage. Then, after osmic acid fixation, ethanol dehydration, and drying, the samples were placed on conductive carbon film double-sided tape and sprayed with gold for 30 s, and the images were captured using a scanning electron microscope.

### Transmission electron microscopy

The MRSA cells after aPDI were washed with PBS and resuspended with electron microscopy fixative. Then the cells were fixed at room temperature for 2 h, and transferred to 4 °C for storage. Then the bacteria underwent agarose pre-embedding, post fixation, dehydration, resin penetration and embedding, polymerization, ultrathin sectioning, and staining. The cuprum grids were observed under TEM and images captured.

### Agarose gel electrophoresis

1 × 10^8^ bacteria were collected in each group, and the treated bacteria were used to extract genomic DNA with a bacterial genomic DNA extraction kit. The extracted DNA was mixed with loading buffer, and then electrophoresed in a 1.5% agarose gel. The electrophoresis buffer was 1×TBE, the voltage was 120 V for 30 min. The agarose gel was placed in a chemiluminometer to detect the DNA bands.

### Sodium dodecyl sulphate–polyacrylamide gel electrophoresis

After aPDI, 2 mL of bacterial suspension at a density of 1.2 × 10^8^ CFU/mL were collected in Eppendorf tubes by centrifugation at 12,000 rpm for 1 min at 4 °C. The supernatant was discarded and each pellet was resuspended in 1 mL PBS. Then each tube was treated with ultrasound at 40 W power for 10 min. The supernatant was collected in Eppendorf tubes by centrifugation at 12,000 rpm for 5 min at 4 °C. The supernatant was mixed with SDS gel-loading buffer. Then the samples were analyzed on 15% SDS-PAGE. After electrophoresis, the gel was stained with Coomassie Brilliant Blue.

### Establishment and aPDI of cutaneous wound infection model in diabetic mice

All animal experiments were performed with a protocol approved by the Animal Care and Use Committees of West China Hospital, Sichuan University (No.20,220,118,002) and were carried out in accordance with the ARRIVE guidelines. Male C57BL/6 mice (SPF, 20–25 g) were purchased from GemPharmatech Co., Ltd (Nanjing, China). All mice were fasted for 12 h before being injected intraperitoneally with 150 mg/kg streptozocin (STZ) solution. Three days later the blood sugar was detected to be > 16.7 mmol/L, confirming the diabetic model was successfully established.

Diabetic mice were randomly divided into four groups (*n* = 10): PBS, RBDA (400 µM) alone, RBDA (400 µM) plus light exposure, or RBDA (400 µM) plus KI (400 mM) and light exposure. Before the skin infection, mice were anesthetized by intraperitoneal injection of 0.3% pentobarbital sodium (0.15 mL/10 g body weight). Then the cutaneous wound infection involved creating a round full-thickness wound with a diameter of 8 mm on the back of anesthetized diabetic mice using a skin biopsy device. Additionally, 50 µL of 1 × 10^6^ CFU/mL MRSA suspension was added to the wound. When the wound became purulent the infection was established.

After anesthetizing the mice as described above, 50 µL of a solution of either 400 µM RBDA or 400 µM RBDA plus 400 mM KI was added dropwise to the wound and incubated for 30 min. Then the surface of the wound was irradiated with 20 J/cm^2^ of 540 nm light, and observations were made daily for the next two weeks until the wound was healed.

### Histological analysis

On the 12th day, mice were sacrificed via CO_2_ (100% CO_2_ at a flow rate of ~ 2 L per min) anesthetization, followed by cervical dislocation to confirm death. The skin tissues at the wound were collected, and then fixed in 4% neutral formalin buffer. After 1 day, the skin samples were embedded in paraffin and cut into 2.5 μm thick slices. The sections were stained with hematoxylin and eosin.

Paraffin embedded sections were deparaffinized by treatment with graded alcohol solutions. Then they were treated by microwave in citric acid (pH 6.0), blocked with 5% goat serum for 30 min, incubated with anti-IL-1β, IL-6, TGF-β, and Ki67 antibodies, followed by incubation with fluorescent labeled secondary antibodies of the corresponding species. The stained slides were observed under a fluorescence microscope, and the results were analyzed with Inform software.

### Statistical analysis

All assays were performed at least in triplicate, and the results are presented as mean ± SD. One-way analysis of variance (ANOVA) for multiple comparisons was performed using SPSS software version 17.0. Values of *P < 0.05, **P < 0.01, ***P < 0.001, and ****P < 0.0001 were considered statistically significant.

## Results

### **Phototoxicity of RBDA-PDI +/- KI on MRSA US300**, ***Escherichia coli*and*Candida albicans in vitro***

**Fig. 1:**
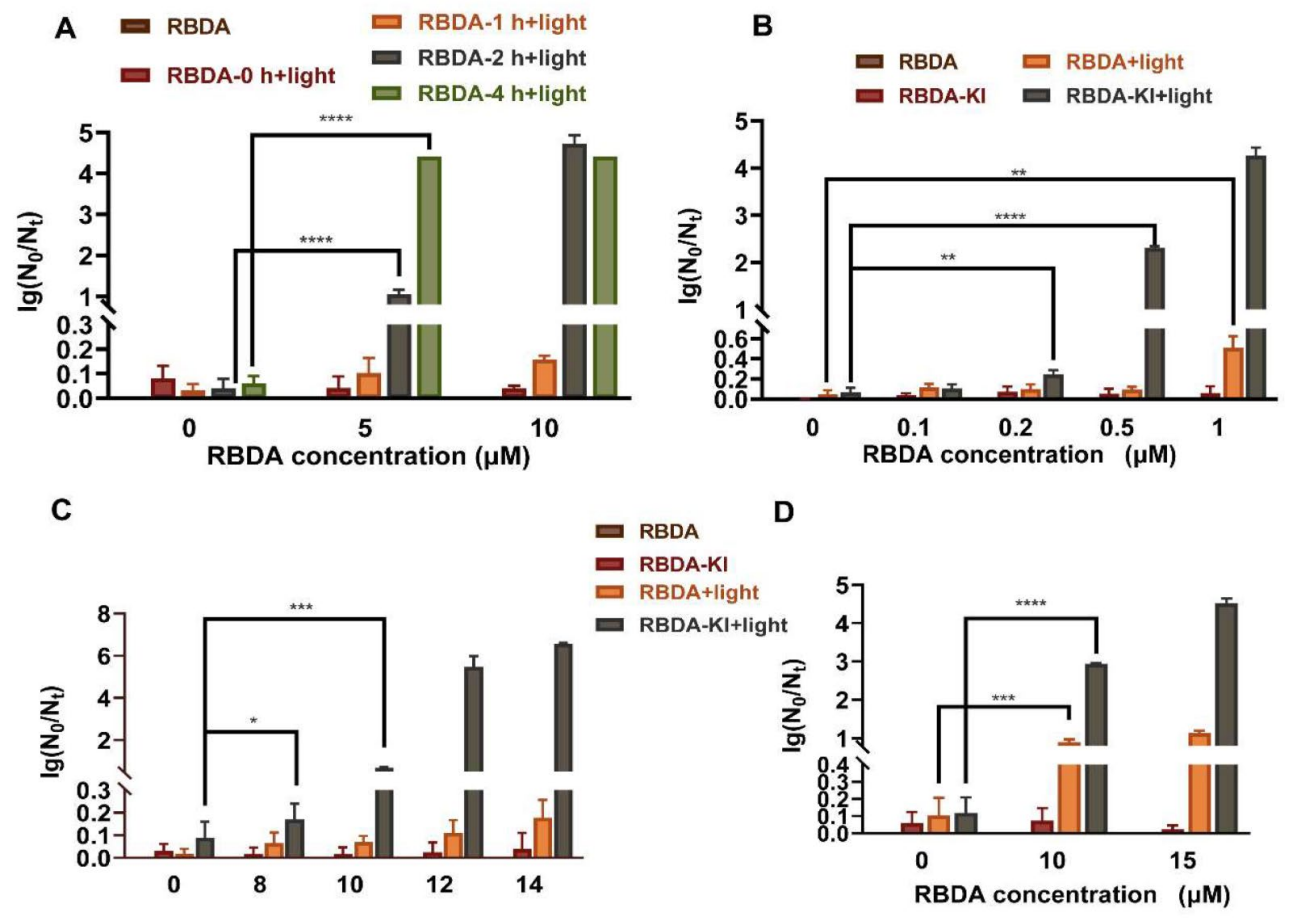
aPDI with RBDA +/- KI in vitro. (**A**) Effect of RBDA on MRSA US300 after incubation during 1 h, 2–4 h followed by 10 J/cm^2^ of 540 nm light. (**B**) Effect of various RBDA concentrations +/- KI on US300 after 2 h incubation and light irradiation. (**C**) Effect of various RBDA concentrations +/- KI on *E. coli* after 2 h incubation and light irradiation. (**D**) Effect of various RBDA concentrations +/- KI on *C. albicans* after 2 h incubation and light irradiation

To explore the optimal incubation time of RBDA with bacteria in vitro, we incubated MRSA with different concentrations of RBDA for different times (1, 2 and 4 h), followed by green light illumination (10 J/cm^2^). The results showed that if MRSA and 10 µM RBDA were incubated for 2 h the aPDI activity was maximized (Fig. 1A), although for 5 µM RBDA 4 h incubation gave the best killing. In contrast, RBDA showed no bactericidal activity under dark conditions. Therefore, a 2 h incubation time was selected for further experiments.

Then we measured the microbial killing with RBDA plus green light, and the effect of adding KI on the number of logs of bacteria or yeasts killed. We selected MRSA US300, *Escherichia coli*, and *Candida albicans* as representatives of Gram-positive bacteria, Gram-negative bacteria and fungi for aPDI experiments. aPDI of all microbial cells was performed after 2 h incubation with RBDA in the dark. The results showed that aPDI of *MRSA*, using RBDA plus 10 J/cm^2^ green light did not show any bactericidal effect up to 1 µM RBDA, but when 100 mM KI was added, 0.2 µM RBDA killed over 2 logs and 1 µM RBDA killed over 4 logs (Fig. 1B). For *E. coli ATCC25922*, there was no significant killing with 14 µM RBDA, but when 100 mM KI was added, 12 µM RBDA killed over 5 logs and 14 µM RBDA killed over 6 logs (Fig. 1C). For *C. albicans SC5314*, 15 µM RBDA showed no significant killing, but when. 100 mM KI was added, 10 µM RBDA killed 3 logs, and 15 µM RBDA killed over 4 logs (Fig. 1D).

These results showed that aPDI using RBDA alone only had a bactericidal effect against MRSA, but not against *E. coli* or *C. albicans*. However, when KI was added, all three microbial species were extensively killed (> 99.9%). Therefore, further research was conducted to clarify the microbicidal mechanism of RBDA-aPDI when combined with KI.

### RBDA aPDI plus KI destroys the cell wall structure of MRSA

**Fig. 2:**
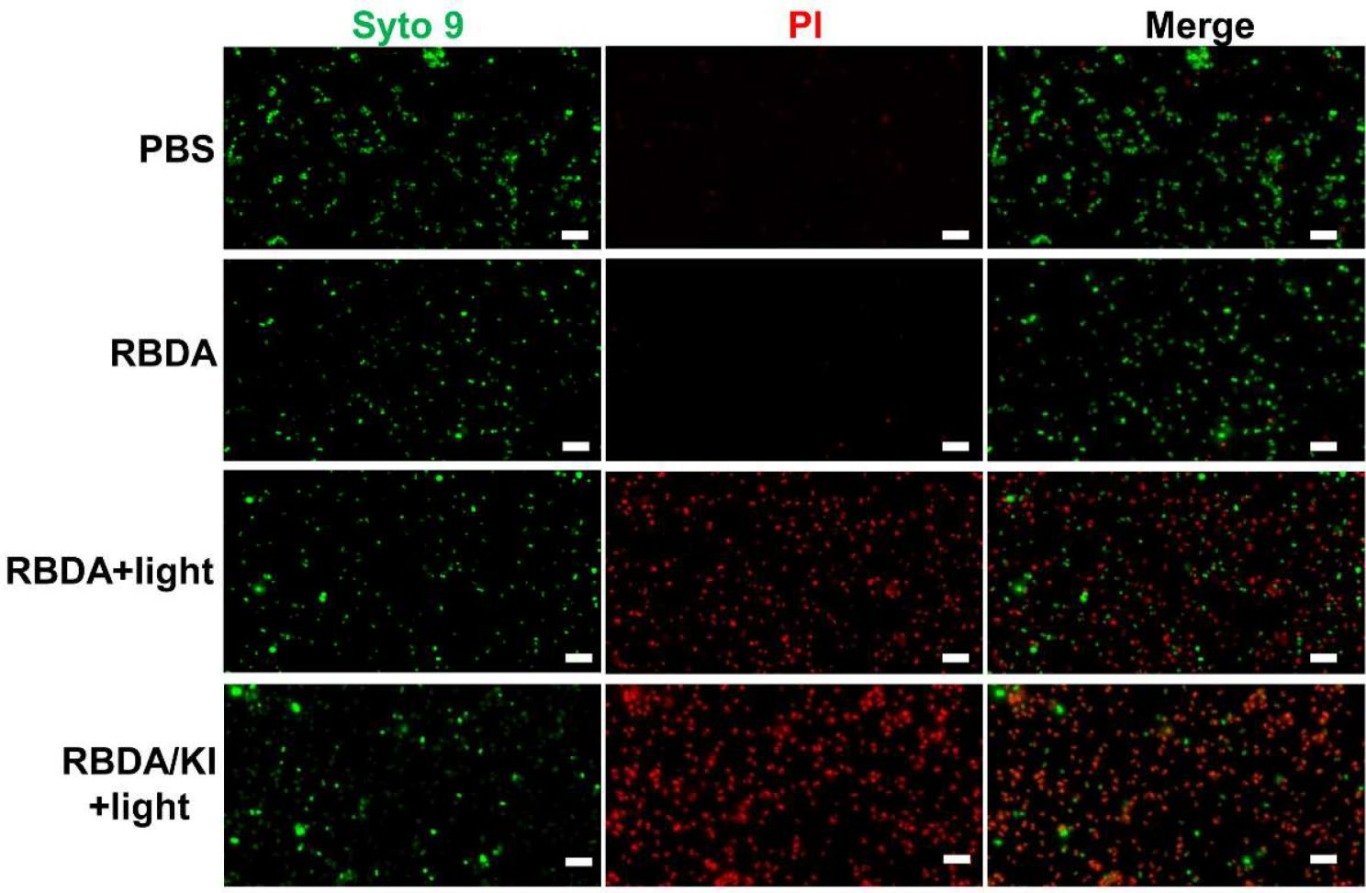
Detection of dead and alive MRSA after in vitro RBDA-aPDI. MRSA was incubated with 1 µM RBDA in the dark for 2 h, followed by 10 J/cm^2^ green light. PI and SYTO9 fluorescent dyes were added to each group of bacteria, and incubated at 37 °C for 15 min in the dark. Green is bacterial DNA bound to SYTO9 in all bacteria. Red is dead bacteria bound to PI

First, we used the Live/Dead™ BacLight™ bacterial viability kit to detect the dead and living bacteria after aPDI (Fig. 2). The PI in this kit can enter the cell through the damaged cell wall to stain the DNA, while the SYTO9 can detect cell viability and the integrity of the cell wall at the same time. Therefore, we treated MRSA with 1 µM RBDA aPDI with or without addition of 100 mM KI, and stained the cells with PI and SYTO9 fluorescent dyes. The results showed that the MRSA after aPDI had a significant number of red cells indicating cell death, and the addition of KI could enhance the number of dead cells.

**Fig. 3:**
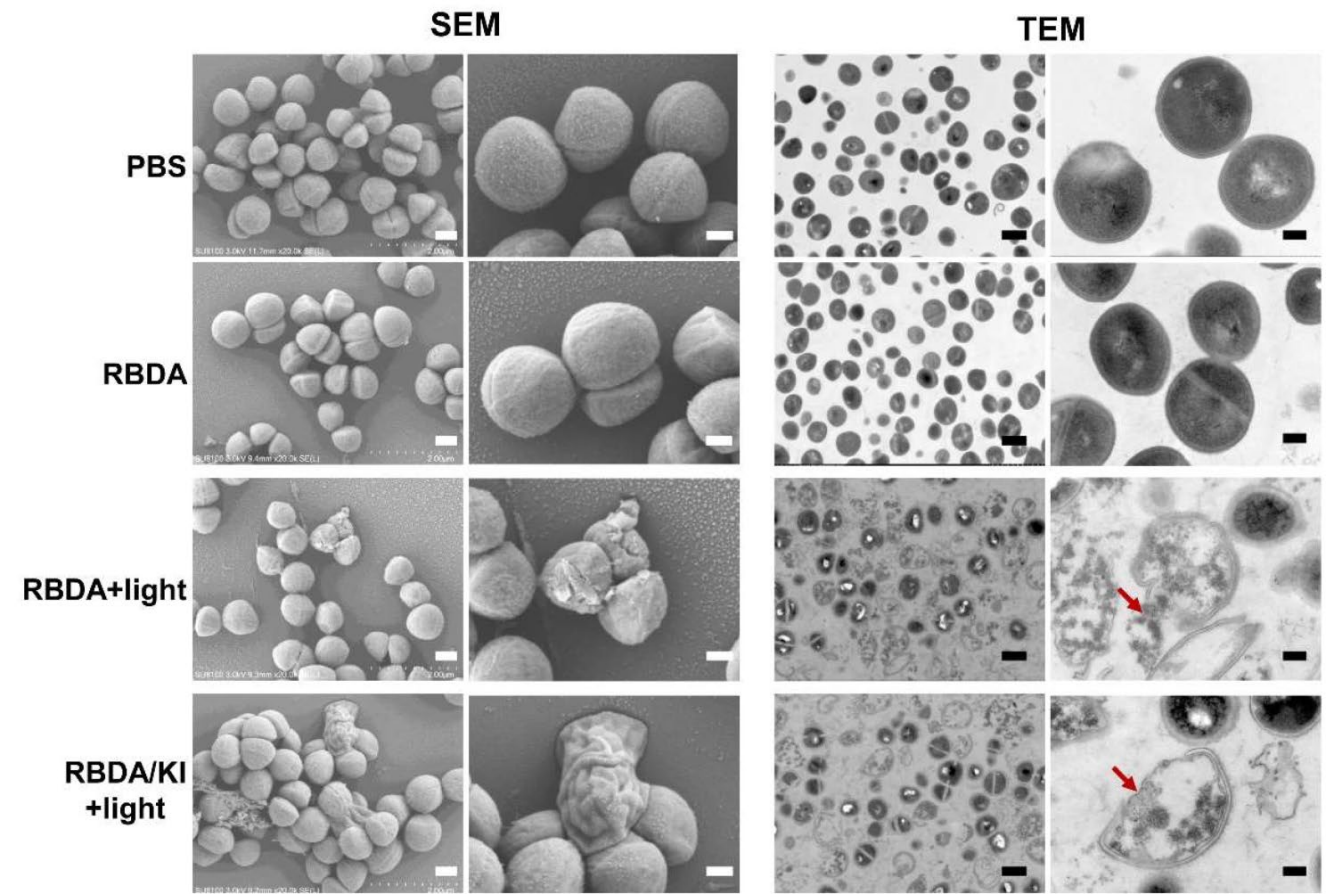
The structure of MRSA cells observed by SEM and TEM. MRSA was incubated with 10 µM RBDA with or without 100 mM KI in the dark for 2 h, followed by 10 J/cm^2^ light. Treated bacteria were incubated overnight at 4 °C and subsequently fixed with 0.25% glutaraldehyde

Moreover, we observed each group of MRSA cells under scanning electron microscopy and transmission electron microscopy (Fig. 3). The results showed that aPDI damaged the cell wall and cell membrane of MRSA cells, led to swelling of the cells and loss of the contents, and finally led to cell death. The addition of KI markedly increased the amount of cell damage as shown by both SEM and TEM.

### Genomic DNA damage and protein degradation in MRSA treated by RBDA-aPDI with or without KI

**Fig. 4:**
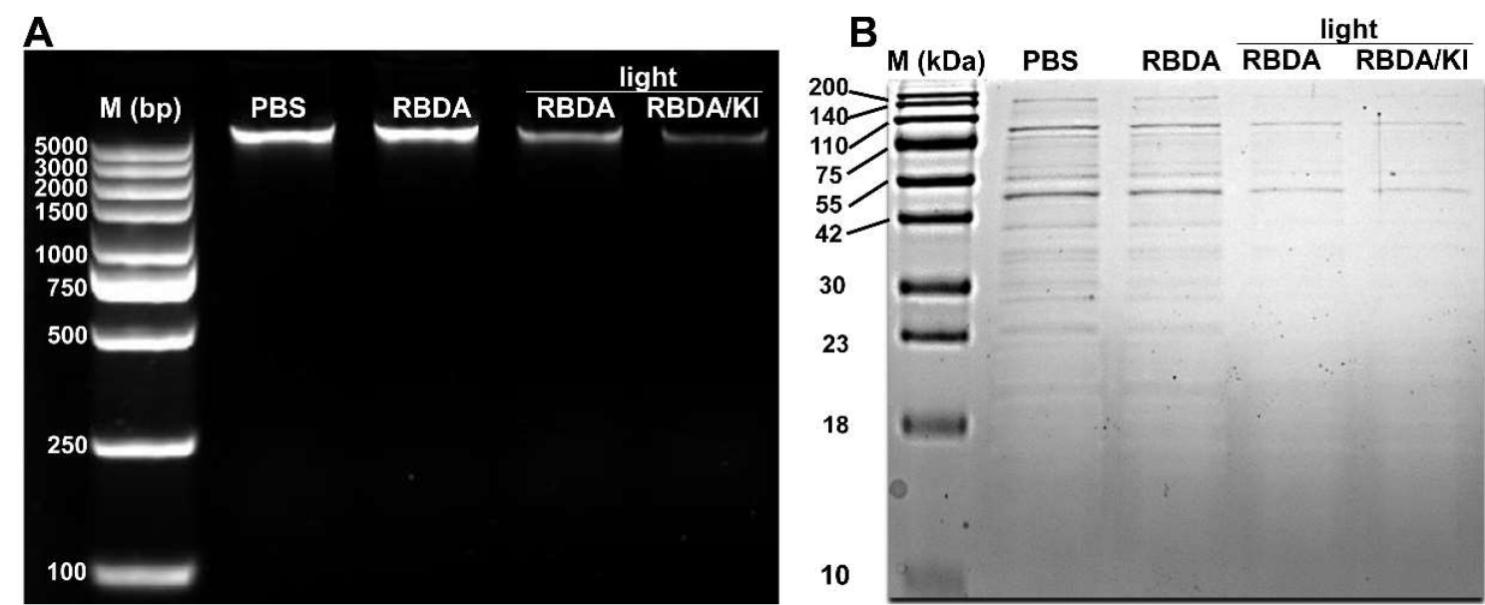
DNA and protein damage in MRSA after aPDI. (**A**) Agarose gel (1.5%) electrophoresis detection of DNA damage. MRSA US300 was incubated with 1 µM RBDA in the dark for 2 h, followed by irradiation (10 J/cm^2^) with or without 100 mM KI. (**B**) SDS-PAGE analysis of protein degradation

We next tested whether aPDI caused DNA damage in MRSA. MRSA cells were treated with 1 µM RBDA with or without 100 mM KI followed by 10 J/cm^2^ green light. The total DNA was extracted by a genomic DNA extraction kit, and the DNA content after aPDI was assessed by agarose gel electrophoresis. The results showed that the DNA band was clearly decreased after RBDA-aPDI and even more so when combined with KI (Fig. 4A). A similar result was obtained with protein degradation as seen in Fig. 4B.

### aPDI with RBDA with or without KI promotes healing of an infected skin wound

**Fig. 5:**
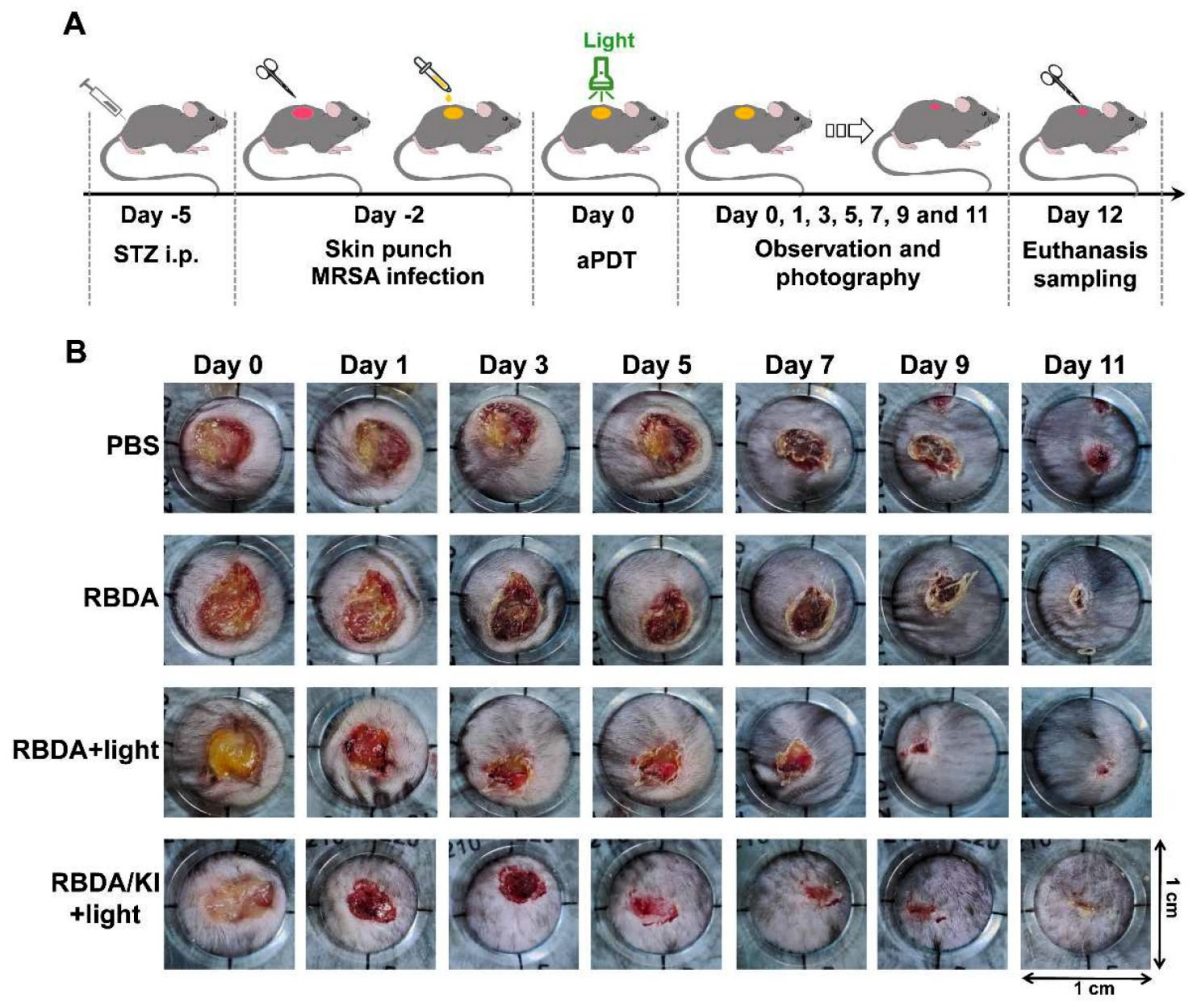
Photodynamic therapy with RBDA +/- KI can promote the healing of infected skin wounds. (**A**) Schematic illustration of MRSA-infected diabetic wound model construction and therapeutic approaches. (**B**) Photographs of the infected wound of diabetic mice in different treatment groups on days 0, 1, 3, 5, 7, 9, and 11 post-treatments. Wounds received 50 µL of RBDA (400 µM) solution with or without 400 mM KI solution for 30 min followed by or not by 20 J/cm^2^ green light. Wound healing was then recorded daily

In order to develop an in vivo wound infection, we first injected STZ into mice to induce a type I diabetes disease state. A skin biopsy was then used to create a full-thickness excision wound on the back of the mice, and the wound was then inoculated with MRSA to form an infection. (Fig. 5A).

The results showed that the amount of pus in the wound after aPDI was rapidly reduced, indicating that the number of bacteria in the wound was lower and the infection was improved. Both the aPDI groups showed markedly faster healing after treatment compared to controls, with the RBDA plus KI group being the fastest (Fig. 5B). In this case the wounds had basically healed by the 10th day.

**Fig. 6:**
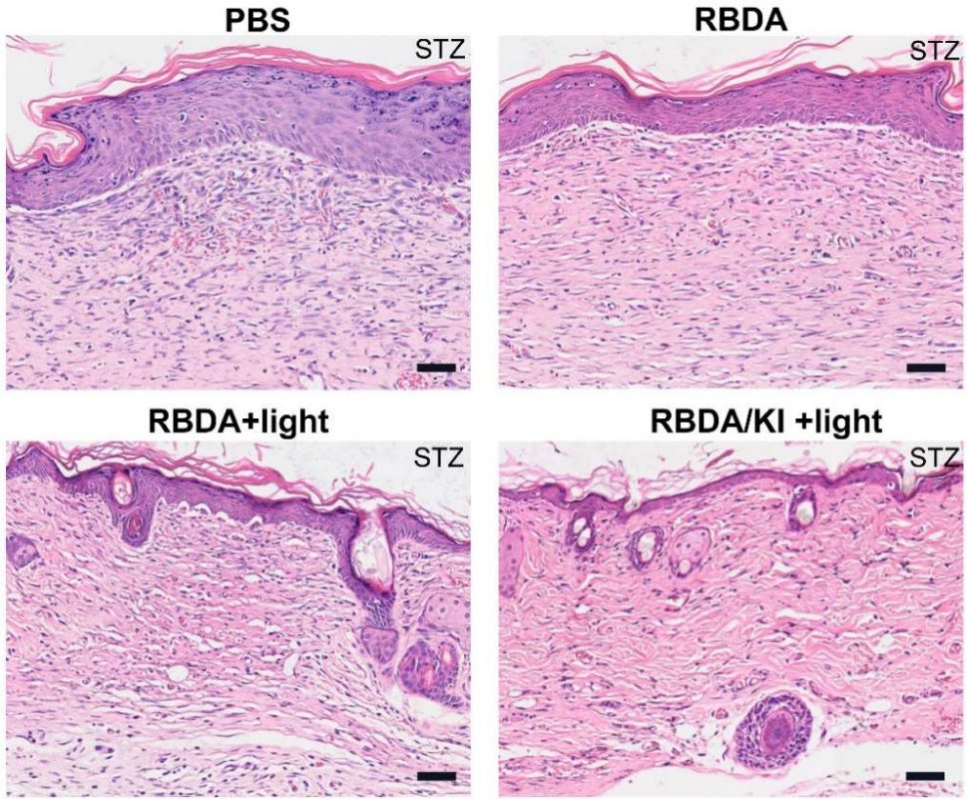
The wound skin tissue samples were taken after 12 days and stained with H&E

The H&E staining of wound tissue on the 12th day showed that due to bacterial infection, a large number of inflammatory cells were still infiltrated in the untreated group and the RBDA control group, while the number of inflammatory cells in both the aPDI groups was significantly reduced. At the same time, the epidermis of the untreated group and RBDA control group was thickened and hyperkeratotic, indicating that it was still in a proliferative state. Adnexa such as hair follicles and secretory glands had not yet been formed. A large number of fibroblasts proliferated in the dermis and secreted type I collagen. In the aPDI groups, the epidermis was slightly keratinized, hair follicles, secretory glands and other appendages had been formed, and the dermis was basically mature (Fig. 6 and Supplementary Figure S1).

Upon treatment with RBDA plus KI and light, inflammation was significantly reduced and skin recovery was found compared to the other groups, including the formation of more hair follicles and thicker collagen. Thus, one session of RBDA plus KI and light could kill MRSA in the mouse infected wound model and efficiently accelerate the recovery.

### aPDI with RBDA can affect the expression of inflammatory factors and proliferation-related proteins in the wound infection model

**Fig. 7:**
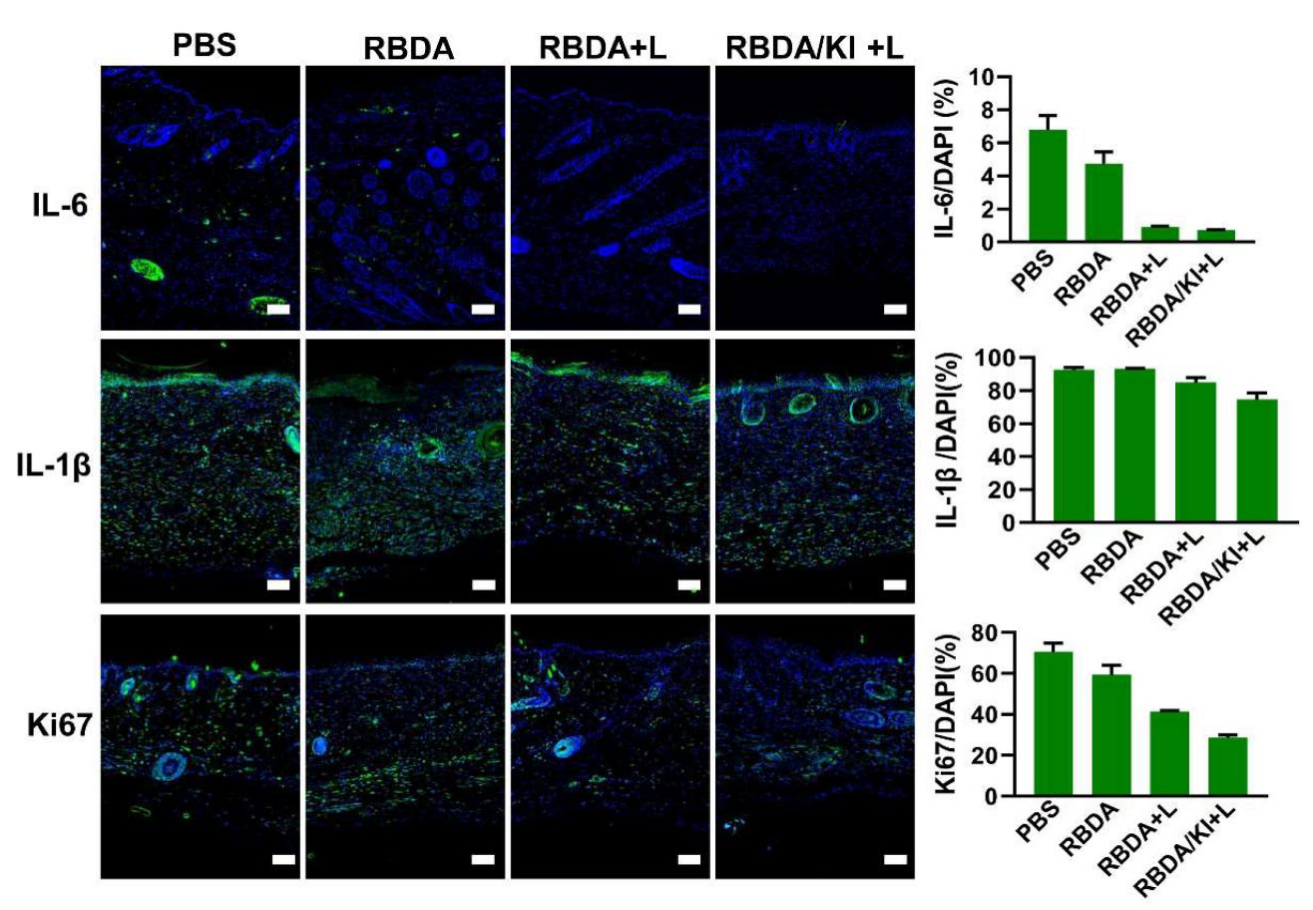
Expression of IL-6, IL-1β and Ki67 in wound tissue samples 12 days after treatment. The tissues were sectioned and stained with antibodies against IL-6, IL-1β and TGF-β. The nuclei were visualized by DAPI co-staining. The scale bar is 100 μm. The bar graphs show the ratio of green antibody fluorescence to blue DAPI fluorescence

To explore how aPDI with RBDA affects markers of healing, we took skin tissue samples on day 12 and measured the expression of IL-6, IL-1β and Ki67 by immunofluorescence (Fig. 7). IL-1β and IL-6 are cytokines that play a central role in the inflammatory response, and in anti-bacterial effects. Among them, IL-6 is highly expressed in the early stage of infection, and is reduced after antibacterial intervention. Ki67 is related to wound healing and is strongly expressed during cellular mitosis.

The results showed that on the 12th day after infection, IL-1β and IL-6 were still highly expressed in the untreated group and the RBDA group, suggesting a strong inflammatory response because the wound was still infected. In the aPDI groups, the expression of IL-1β was still observed, but IL-6 was almost not expressed, indicating that the bacterial infection had been controlled after aPDI. The expression of Ki67 was high in the untreated group and the RBDA group indicated that the cells were still proliferating at 12 days. However, in the aPDI groups, Ki67 was much lower because the wounds had basically healed.

## Discussion

Our previous publication showed that the well-known xanthene dye RB could act as a highly efficient PS against Gram-positive bacteria (MRSA) when excited by green light [10]. However, its effect on killing Gram-negative bacteria (*E. coli* and *Pseudomonas aeruginosa)* or yeast *(C. albicans)* was very poor. We had previously shown that several photosensitizers including Photofrin [15], which were already known to be ineffective against Gram-negative bacteria, could provide dramatically potentiated light-mediated microbial killing by addition of the simple non-toxic inorganic salt KI [16]. Therefore, we tested this approach with RB plus KI. If 100 mM KI was added to 10 µM RB there was a large increase in microbial killing (6 logs more) of *E. coli* and *C. albicans* [10] after green light exposure. Although the killing of Gram-positive MRSA was potentiated by addition of KI, the difference was not so impressive because RB was highly effective as a photosensitizer on its own.

The photochemical mechanism of RB has been found to operate largely via the type II pathway involving energy transfer from the long-lived RB triplet state to ground-state triplet oxygen to produce reactive singlet oxygen [17]. The number of heavy halogen atoms (4 iodine and 4 chlorine atoms) in the molecule means that RB has a singlet oxygen quantum yield of about 0.86 [18]. In fact, RB is often used as a standard in determinations of singlet oxygen quantum yields [19]. The action mechanism of KI potentiation of the aPDI effect has been investigated, and it was found that the singlet oxygen created by light excitation of RB could oxidize iodide anions to form peroxyiodide anions [20]. Peroxyiodide is an unstable species and can break down via two different pathways [21]. The first breakdown reaction produces free molecular iodine and hydrogen peroxide, both of which are fairly stable but intrinsically antimicrobial substances. The second breakdown pathway involves production of a pair of radicals (I_2_^•−^ and HOO^•^) both of which are highly reactive short-lived species, and can damage microbial cells. In order for these reactions to take place, the short-lived singlet oxygen must be able to come into close contact with the iodide anions. In the case of RB, which does not easily penetrate into Gram-negative bacterial or fungal cells, the RB will exist in the extracellular aqueous medium in the same location as the iodide anions, which also do not penetrate into microbial cells.

The situation is different when RBDA is employed as the PS, because this compound must first penetrate into the microbial cells in order to be hydrolyzed to form the photochemically active PS, free RB. However, once the RB has been formed inside the bacterial cells, it could then diffuse outside again into the aqueous medium. This might explain why a long incubation time of 2 h was necessary to obtain the optimum levels of bacterial killing. If the RB has diffused outside the microbial cells, it will be able to generate singlet oxygen in the same location as the iodide anions, thus allowing the generation of the bactericidal iodine species as described above. The need for the iodide anions to be able to trap the short-lived singlet oxygen molecules explains why a fairly high concentration of 100 mM was required, and an even higher concentration of 400 mM was needed in vivo.

The use of Rose Bengal acetate (RBAc) as an enzyme-activated PS was first described in 1997 by Botiroli et al. [22]. These investigators studied the balance between the uptake and hydrolysis of RBAc and the efflux of RB in rat glioma cells in vitro. Since that first report, several groups in Italy have looked at RBAc-mediated PDT to trigger apoptosis [23], autophagy [24], etc. [13] in HeLa human cancer cells. There has been one report of RBAc being exploited to produce aPDI bacterial killing [14]. Manoil and co-workers used the Gram-positive bacteria *Enterococcus faecalis* and incubated the cells with 200 µM RBAc for up to 900 min before exposure to blue light resulting in at least 4 logs of killing [14]. It should be noted that the conditions employed by Manoil et al. [14] (200 µM RBAc for 900 min) were much higher than used in the present study (15 µM RBAc for 120 min).

In the present study although RBDA alone was highly active against Gram-positive MRSA, it was not as active as free RB, which we previously showed could kill three logs of bacteria at only 100 nM concentration, and completely eradicate the cells when used at 200 nM or higher with 20 J/cm^2^ green light [10]. Moreover, to our knowledge, the present study is the first time that RBDA has been reported to mediate aPDI of Gram-negative bacteria and fungal yeast.

We used a model of an excisional wound infected with MRSA in diabetic mice. Although MRSA can be highly pathogenic and virulent in humans [25], in our experience this is not necessarily the case in mice [26]. If MRSA bacteria are introduced into a clean excisional wound created in healthy mice, an established infection can be difficult to obtain, Therefore investigators have often employed diabetic mice, such as the mutant db/db mice reported by Shi et al. [27], or the streptozotocin-injected mice we employed in the present study. The use of diabetic mice reflects the human situation, where MRSA wound infections in diabetic patients are 5 times more common than in non-diabetic individuals [28]. In our study we found that aPDI mediated by RBDA and green light stimulated wound healing in the MRSA infected wounds in diabetic mice. This result was highly encouraging because it might have been supposed that a lipophilic compound such as RBDA would be taken up into the host cells inside the wound, and this would lead to substantial phototoxicity after green light irradiation, which would have been expected to inhibit wound healing. Future investigations could employ immunohistochemical techniques to see whether there is any evidence of apoptosis or autophagy occurring in the host cells within the wound, as reported in vitro by Panzarini et al. [29]. It should be noted that we did not measure the bacterial burden of living microbial cells inside the mouse wounds, either by sampling and quantification of the number of CFUs [30], or by optical imaging of bioluminescent bacteria in living mice [31] which allows longitudinal monitoring of individual animals and better statistical analysis [32]. Therefore, it is difficult to tell whether the beneficial effects of aPDI applied to the infected wounds, could be attributed to killing of the actual MRSA bacterial cells, or to some PDT effects on the host cells. These host cell effects could involve stimulation of healing mechanisms, such as cellular proliferation, migration, angiogenesis and collagen synthesis [33, 34]. An alternative possibility is that the PDT could stimulate the innate immune response involving bactericidal neutrophils within the wounds, which could then attack and kill the MRSA bacteria, as we reported previously in a mouse model of bacterial arthritis [35].

It was possible to observe a trend for the addition of KI being able to potentiate the effectiveness of RBDA-aPDI to stimulate wound healing (Fig. 5), although no statistical comparisons could be carried out. The histological and immunohistochemistry results also supported some additional benefits of adding KI into the RBDA-mediated PDT of the infected wounds.

## Conclusions

We have shown that RBDA plus green light can be used to kill both Gram-positive and Gram-negative bacteria as well as fungal yeast, and that this killing can be strongly potentiated by the addition of the non-toxic salt KI. The addition of KI could provide some additional benefits on the healing of MRSA infected excisional wounds treated with RBDA-mediated aPDI in a diabetic mouse model. The addition of non-toxic KI deserves to be tested in clinical trials of aPDI for infected wounds in patients [36].

### Electronic supplementary material

Below is the link to the electronic supplementary material.

Supplementary Material 1

## Data Availability

The datasets used and/or analyzed during the current study are available from the corresponding author on reasonable request.
