# Supplementary material for: Rose Bengal diacetate-mediated antimicrobial photodynamic inactivation: potentiation by potassium iodide and acceleration of wound healing in MRSA-infected diabetic mice
Source: BMC Microbiol. 2024 Jul 5;24:246. doi: 10.1186/s12866-024-03401-6 (PMC11225387; doi:10.1186/s12866-024-03401-6)
Supplement: Supplementary file 1 — Supplementary Material 1 [file 12866_2024_3401_MOESM1_ESM.docx]

Support Information

Rose Bengal diacetate-mediated antimicrobial photodynamic inactivation: potentiation by potassium iodide and acceleration of wound healing in MRSA-infected diabetic mice

Danfeng Wei^1,2^, Michael R Hamblin^3^, Hao Wang^1,2^, Reza Fekrazad^4,5^, Chengshi Wang^6^, Xiang Wen^1,2*^

1. Department of Dermatology, West China Hospital, Sichuan University, Chengdu 610041, China.

2. Laboratory of Dermatology, Clinical Institute of Inflammation and Immunology, Frontiers Science Center for Disease-related Molecular Network West China Hospital, Sichuan University, Chengdu 610041, China.

3. Laser Research Centre, Faculty of Health Science, University of Johannesburg, Doornfontein 2028, South Africa

4. Radiation Sciences Research Center, Laser Research Center in Medical Sciences, AJA University of Medical Sciences, Tehran, Iran.

5. International Network for Photo Medicine and Photo Dynamic Therapy (INPMPDT), Universal Scientific Education and Research Network (USERN), Tehran, Iran.

6. Department of Endocrinology and Metabolism, Center for Diabetes and Metabolism Research, West China Hospital, Sichuan University, Chengdu 610041, China

* Correspondence: Corresponding Author: Xiang Wen, xiangwen_wcums@163.com


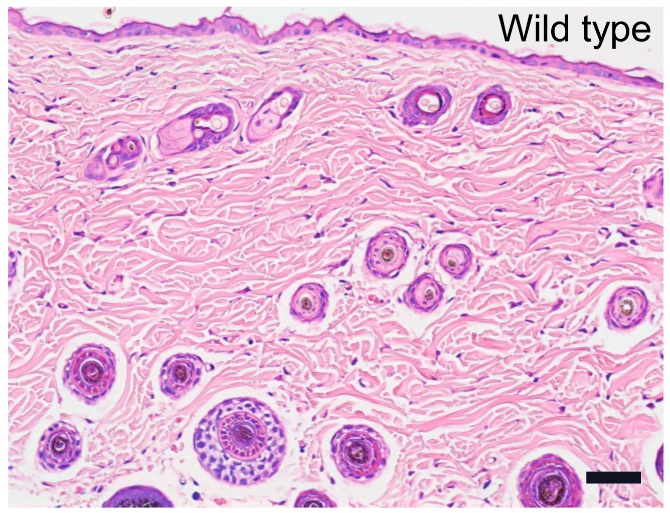


**Figure S1. The H&E staining image of skin tissue of wild type C57BL/6 mouse.** C57BL/6 mouse established diabetes model according to the method in 2.9. The back tissue was taken after 12 days of feeding under the same conditions as other groups. HE staining was performed after paraffin embedding.
